# miR-145 supports cancer cell survival and shows association with DDR genes, methylation pattern, and epithelial to mesenchymal transition

**DOI:** 10.1186/s12935-019-0933-8

**Published:** 2019-09-06

**Authors:** Siddharth Manvati, Kailash Chandra Mangalhara, Ponnuswamy Kalaiarasan, Rupali Chopra, Gaurav Agarwal, Rakesh Kumar, Sunil Kumar Saini, Monika Kaushik, Ankita Arora, Usha Kumari, Rameshwar Nath Koul Bamezai, Pawan Kumar Dhar

**Affiliations:** 1 School of Biotechnology, Jawaharlal Nehru University, New Delhi, India; 2 National Centre of Applied Human Genetics, School of Life Sciences, Jawaharlal Nehru University, New Delhi, India; 3 Sanjay Gandhi Postgraduate Institute of Medical Sciences, Lucknow, Uttar Pradesh, India; 4 School of Biotechnology, Shri Mata Vaishno Devi University, Kakryal, Katra, Jammu and Kashmir India; 5 Faculty of Medicine, AIMST University, Bedong, Malaysia

**Keywords:** miR-145, SMAD3, BRCA2, DR5, Epithelial–mesenchymal transition, Vimentin, Methylation

## Abstract

**Background:**

Despite several reports describing the dual role of miR-145 as an oncogene and a tumor suppressor in cancer, not much has been resolved and understood.

**Method:**

In this study, the potential targets of miR-145 were identified bio-informatically using different target prediction tools. The identified target genes were validated in vitro by dual luciferase assay. Wound healing and soft agar colony assay assessed cell proliferation and migration. miR-145 expression level was measured quantitatively by RT-PCR at different stages of breast tumor. Western blot was used to verify the role of miR-145 in EMT transition using key marker proteins.

**Result:**

Wound healing and soft agar colony assays, using miR-145 over-expressing stably transfected MCF7 cells, unraveled its role as a pro-proliferation candidate in cancerous cells. The association between miR-145 over-expression and differential methylation patterns in representative target genes (*DR5*, *BCL2*, *TP53*, *RNF8*, *TIP60*, *CHK2*, and *DCR2*) supported the inference drawn. These in vitro observations were validated in a representative set of nodal positive tumors of stage 3 and 4 depicting higher miR-145 expression as compared to early stages. Further, the role of miR-145 in epithelial–mesenchymal (EMT) transition found support through the observation of two key markers, Vimentin and *ALDL*, where a positive correlation with Vimentin protein and a negative correlation with *ALDL* mRNA expression were observed.

**Conclusion:**

Our results demonstrate miR-145 as a pro-cancerous candidate, evident from the phenotypes of aggressive cellular proliferation, epithelial to mesenchymal transition, hypermethylation of CpG sites in DDR and apoptotic genes and upregulation of miR-145 in later stages of tumor tissues.

## Background

MicroRNAs are small non-coding RNA molecules with a potential to regulate the cellular machinery directly or indirectly. Functions of most of the ~ 1900 mature miRNA identified till date remain to be understood properly. miR-145 is one such candidate reported to play a dual role, of a tumor suppressor by targeting the expression of, FLI1 [1, 2], TIRIP [3], SOX9 [4], VEGF [5], Ets 1 [6], YES, STAT1 [7], p70S6K1 [8], Dab2 [9], PAK4 [10], JAM-A [11], CLDN10 [12] FSCN1 [13], RTKN [14] genes; and in the neointimal formation of vascular smooth muscle cells [15]. miR-145 has also been reported to facilitate ES cell differentiation by repressing the core pluripotency factor, OCT4 [16].

The expression of miR-145 has been reported to be down-regulated in cervical [17], hepatic [18] and hepatocellular [19], colorectal [20, 21], serous ovarian [22], ovarian [23], colon [24], oral [25], prostate [26], gastric [27], bladder [28], nasopharyngeal [29], lung [30], ACTH-secreting pituitary [31], B-CLL (B cell chronic lymphocytic leukemia) [32] and breast [33] cancers, dedifferentiated VSMCs and balloon-injured arteries [34], acute and chronic vascular stress [35], myelodysplastic syndrome [36]. Whereas, in pancreatic ductal adenocarcinoma [32], polycythemia vera [37], multiple sclerosis [38], the expression of miR-145 was found to be up-regulated. Suggestions of miR-145 playing a role in the development of colon and rectal cancers but not in its progression [39] have been made, thus raising a question about its exact role in tumour initiation and development. It was, therefore, pertinent to understand the dual role of miR-145 in cancer, using both in vitro and cellular assays.

## Results

### miR-145 down-regulates mRNA expression of SMAD3, DR5, BRCA2

**Fig. 1:**
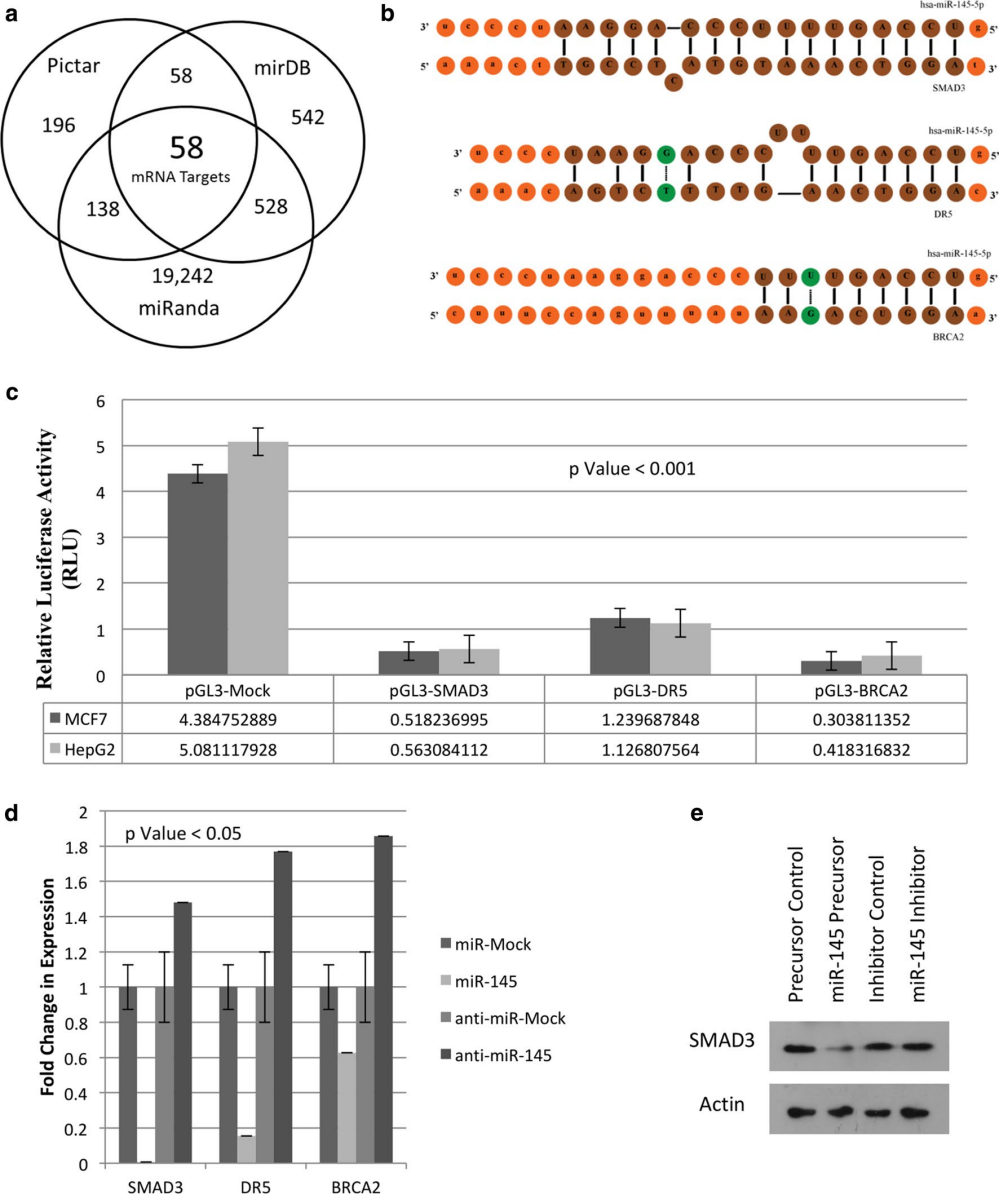
SMAD3, DR5 and BRCA2 as the novel targets of miR-145: **a** Predicted 58 common targets for miR-145 in all the three tools (PicTaR, mirDB and miRanda), which included SMAD3, DR5 and BRCA2 with highest score; **b** binding sites of miR-145 identified at the 3′UTR region of the three targets, SMAD3, DR5 and BRCA2; **c** *Luc* gene activity showing a significant decrease when miR-145 was over-expressed in cells expressing Luc with 3′UTR of SMAD3 or DR5 or BRCA2, in MCF7 and HepG2 cells; **d** real-time PCR using Sybergreen, showing a significant decrease and increase in fold change of mRNA expression of identified targets (SMAD3, DR5 and BRCA2) in MCF7 cells under miR-145 and anti-miR-145 transfected conditions, respectively. **e** Western blotting of SMAD3 in presence and absence of miR-145 in MDAMB231 cell line

The targets of miR-145 were predicted using bio-informatics tools. Of 19,980 predicted 3′UTR targets (PicTar: 196; mirDB: 542; miRanda: 19,242), 58 emerged common amongst all the three tools used (Fig. 1a), showing SMAD3, DR5, BRCA2 with highest prediction scores. Validation of the presence of miR-145 binding site at 3′UTR (Fig. 1b) in the three targets using luciferase reporter assay, showed a significantly decreased activity in presence of exogenous miR-145 in MCF7 (SMAD3: 0.51; DR5: 1.23; BRCA2:0.30) and HepG2 (SMAD3:0.51; DR5: 1.23; BRCA2: 0.30) cells, when compared with the activity of luciferase reporter without miR binding site (MCF7: 4.38; HepG2: 5.08) (Fig. 1c).

Further validation of the three targets, through their cellular status in MCF7 cells with over-expressing miR-145, exhibited decreased expression of SMAD3; DR5; BRCA2 (0.04-fold; 0.1-fold; 0.6-fold) when compared to the control (pEP-miR-Mock considered as onefold). Inhibition with anti-miR-145, resulting in a reversal and an increased expression in cells of the three target genes (1.5-fold; 1.7-fold; 1.9-fold) as compared to mock control (anti-miR-mock considered as onefold) confirmed that miR-145 targeted these three targets (Fig. 1d). The protein expression of one of the novel targets SMAD3, performed using Western Blotting was observed to be downregulated in presence of miR-145 precursors as compared to control mimics (Fig. 1e).

### miR-145 over-expression in late stage tumour tissues correlates with apoptotic and DDR gene methylation in vitro

**Fig. 2:**
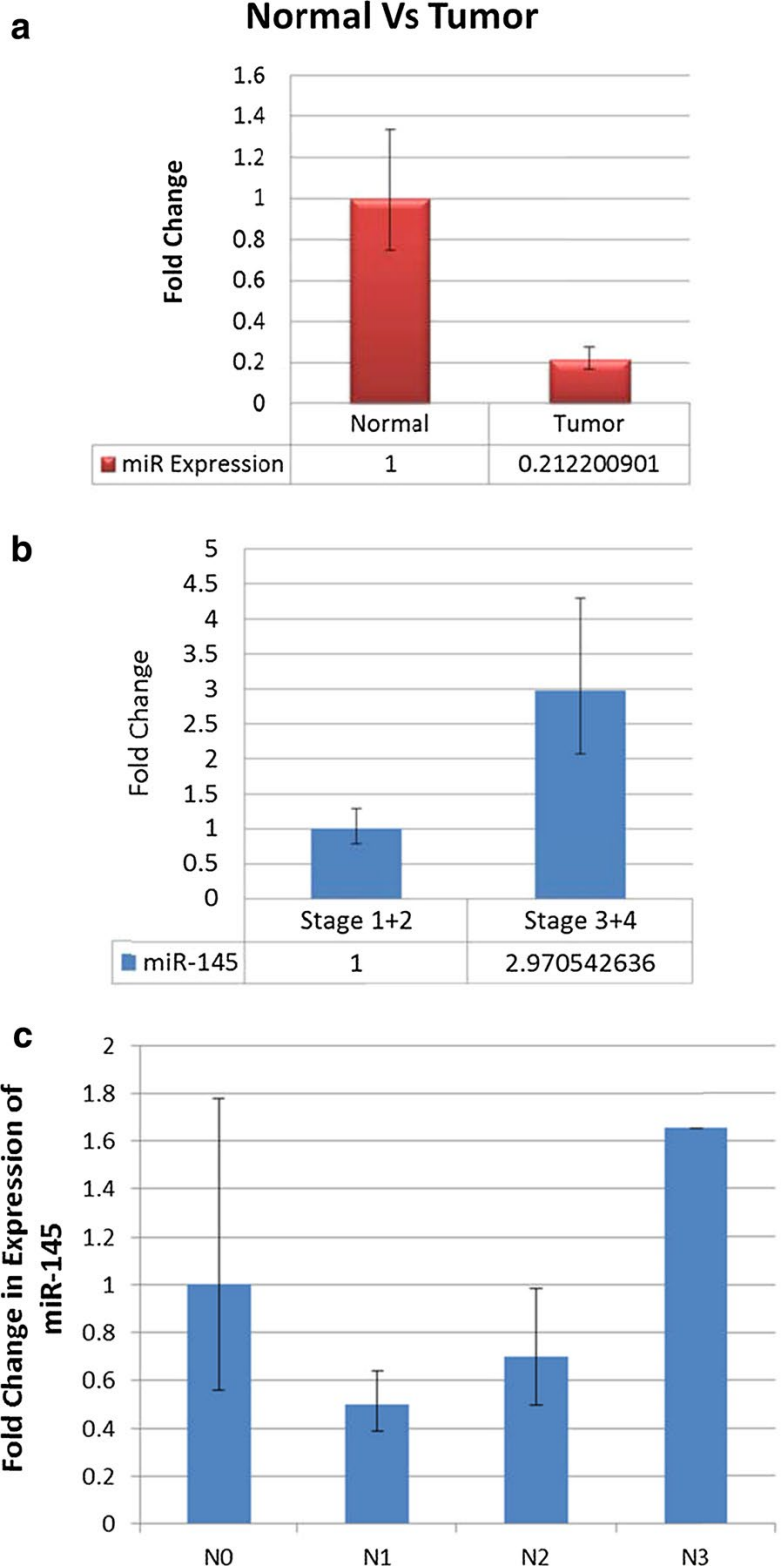
Expression status of miR-145: in **a** normal vs tumor breast cancer samples (p value < 0.01); **b** tumor samples grouped in stage 1 + 2 and stage 3 + 4 (p value < 0.001) and; **c** tumor samples grouped on the basis of number of nodes getting effected (p value < 0.05)

In-vivo studies in a representative set of 72 (36 pairs) samples showed down-regulated expression of miR-145 in sporadic breast tumours when compared to adjoining normal tissue (Fig. 2a). However, the stage wise analysis revealed that the expression of miR-145 increased significantly in tumour samples grouped together for stage 3 + stage 4 as compared to tumour samples belonging to stage 1 + stage 2 (Fig. 2b). Interestingly, miR-145 expression showed an increase with the number of nodes involved (Fig. 2c) with a concomitant differential methylation pattern of most of the studied CpG positions in apoptotic and DDR genes (*DR5*, *BCL2*, *TP53*, *RNF8*, *TIP60*, *CHK2*, *DCR2*), both in vivo and in vitro experiments (Additional file 1: Table S1).

**Fig. 3:**
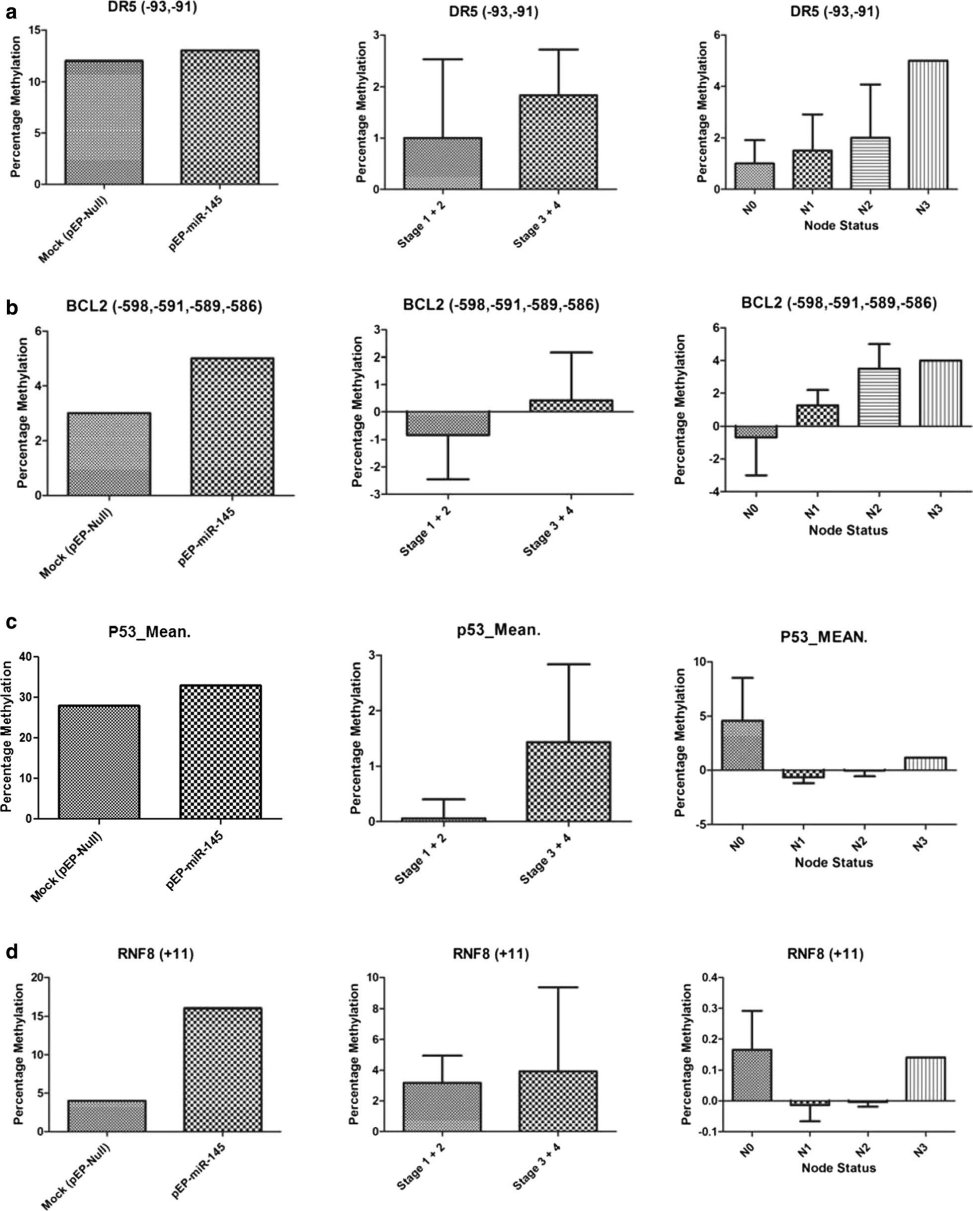

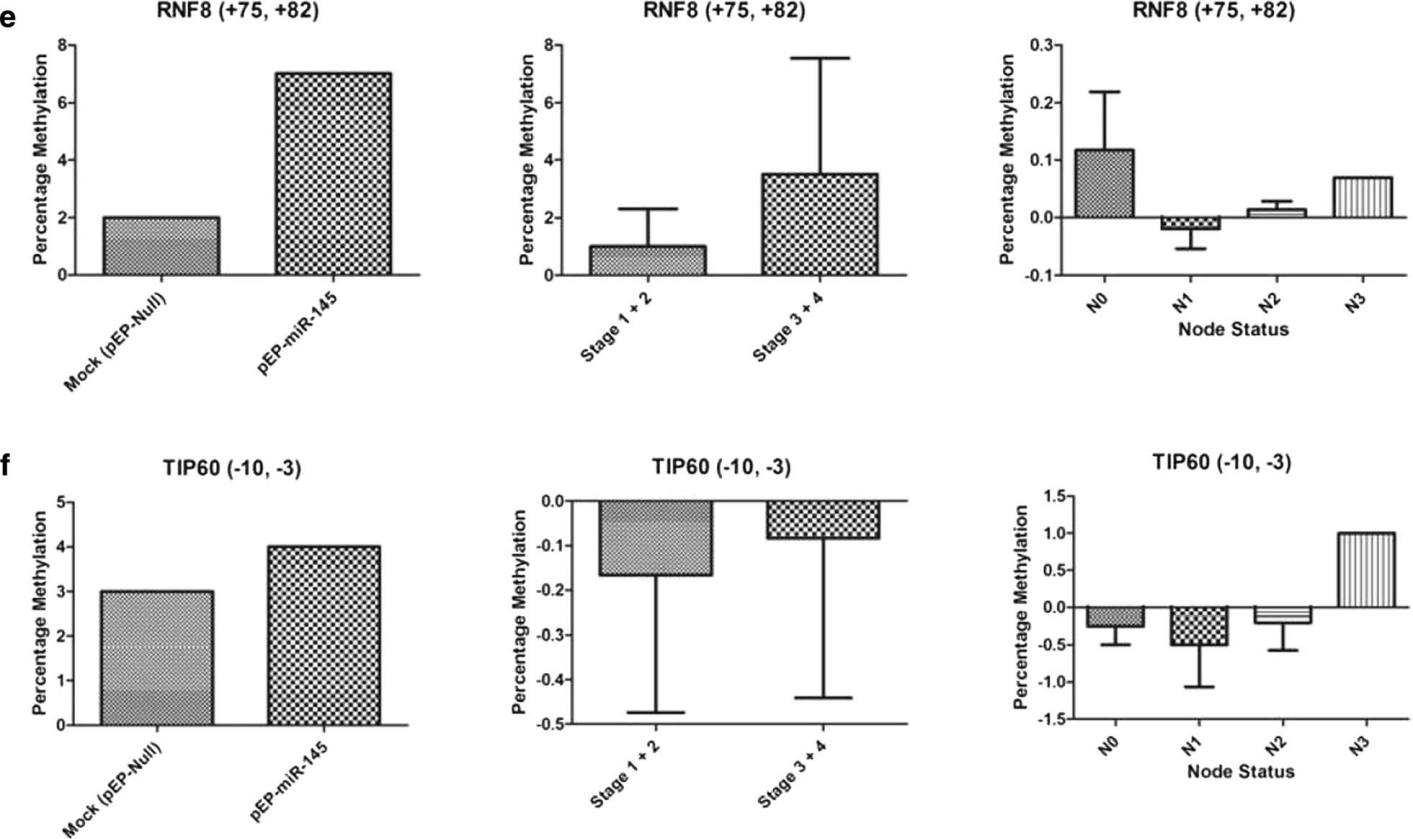
Increasing methylation pattern: observed at different CpG positions within the gene **a** *DR5* (− 93, − 91), **b** *BCL2* (− 598, − 591, − 589, − 586), **c** *TP53* (− 78, − 75, − 22, − 15, + 91, + 158, + 171, + 175), **d** *RNF8* (+ 11), **e** *RNF8* (+ 75, + 82) and **f** *TIP60* (− 10, − 3) when compared in miR-145 over-expressing stable cells, tumor stage wise groups and node status of breast cancer samples

**Fig. 4:**
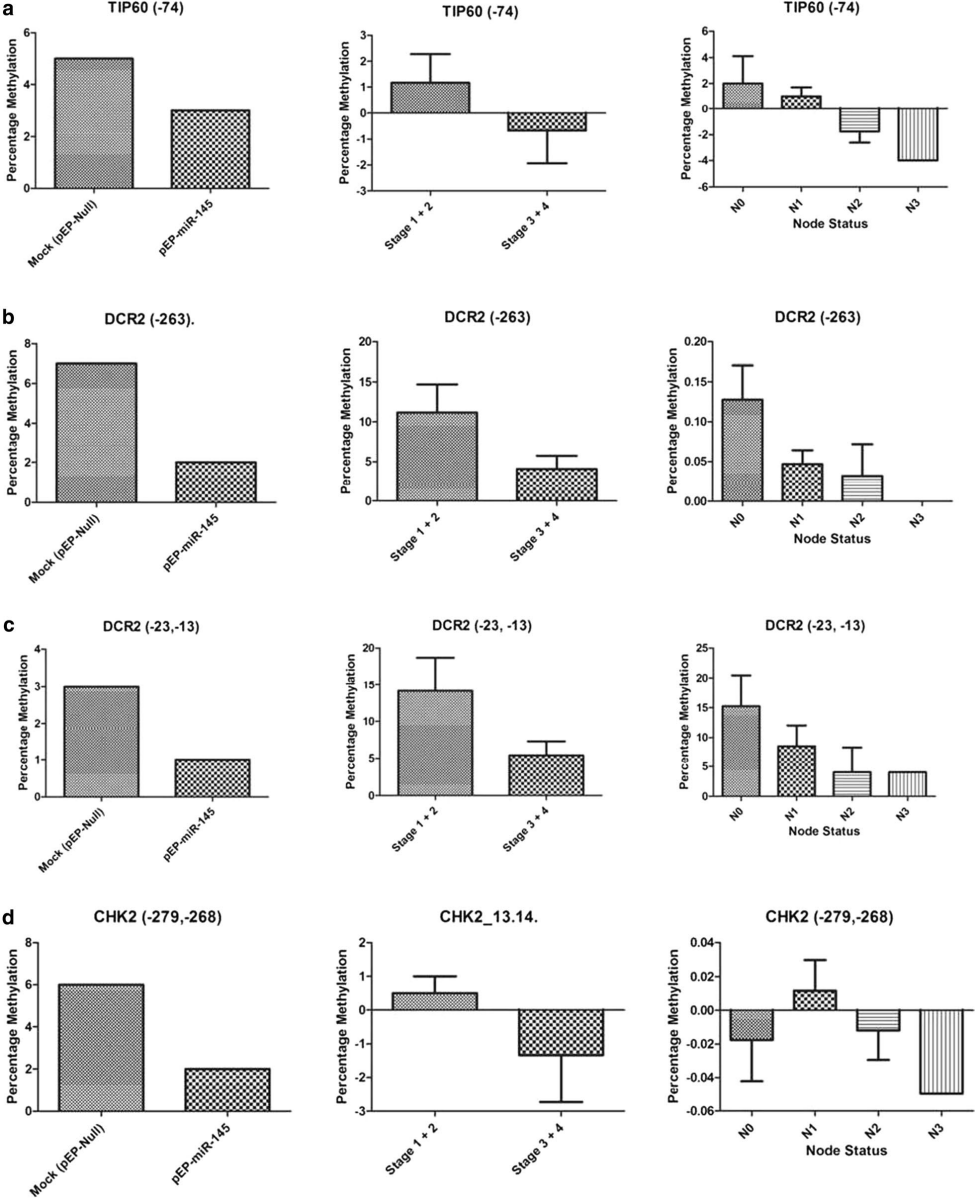
Decreasing methylation pattern: Observed at different CpG positions within the gene **a** TIP60 (− 74), **b** DCR2 (− 263), −  DCR2 (− 23 and − 13), and **d** *CHK2* (− 279, − 268) when compared in miR-145 over-expressing stable cells, tumor stage wise groups and node status of breast cancer samples

The methylation status of CpG positions of genes related to cell death and survival [40] was assessed in vitro in miR-145 stably transfected and over-expressing MCF-7 cells and validated in vivo in a representative set of sporadic breast cancer tissues (Figs. 3, 4; Additional file 1: Table S1). The methylation status of DR5 CpG positions (− 93, − 91) revealed increased average percentage methylation (stage 1 + 2: 2%; stage 3 + 4: 2.88%) in tumors, correlating with the observations made in vitro. Further, DR5 methylation at CpG position (− 93, − 91) showed an increased methylation with the number of nodes involved (N0: 1.00%; N1: 1.5%; N2: 2.00%; N3: 5.00%), supporting what was observed in in vitro between DR5 methylation/regulation and experiments with miR-145 over-expression. A simultaneous hypermethylation of *BCL2*, *TP53*, *RNF8* and hypomethylation of *DCR2*, *CHK2* observed in vitro and in vivo studies supported the assessment of pro-proliferative role of miR-145.

Since a positive association between DNA damage response with epithelial to mesenchymal transition [41] and induction of methylation [40] has been reported, it was pertinent to adjudge our conclusions on EMT by confirming the association of miR-145, if any, with chosen DDR genes both in miR-145 over-expressing MCF-7 cells and in sporadic breast cancer tissues.

### miR-145 over-expression supports aggressive proliferation and epithelial to mesenchymal transition (EMT)

**Fig. 5:**
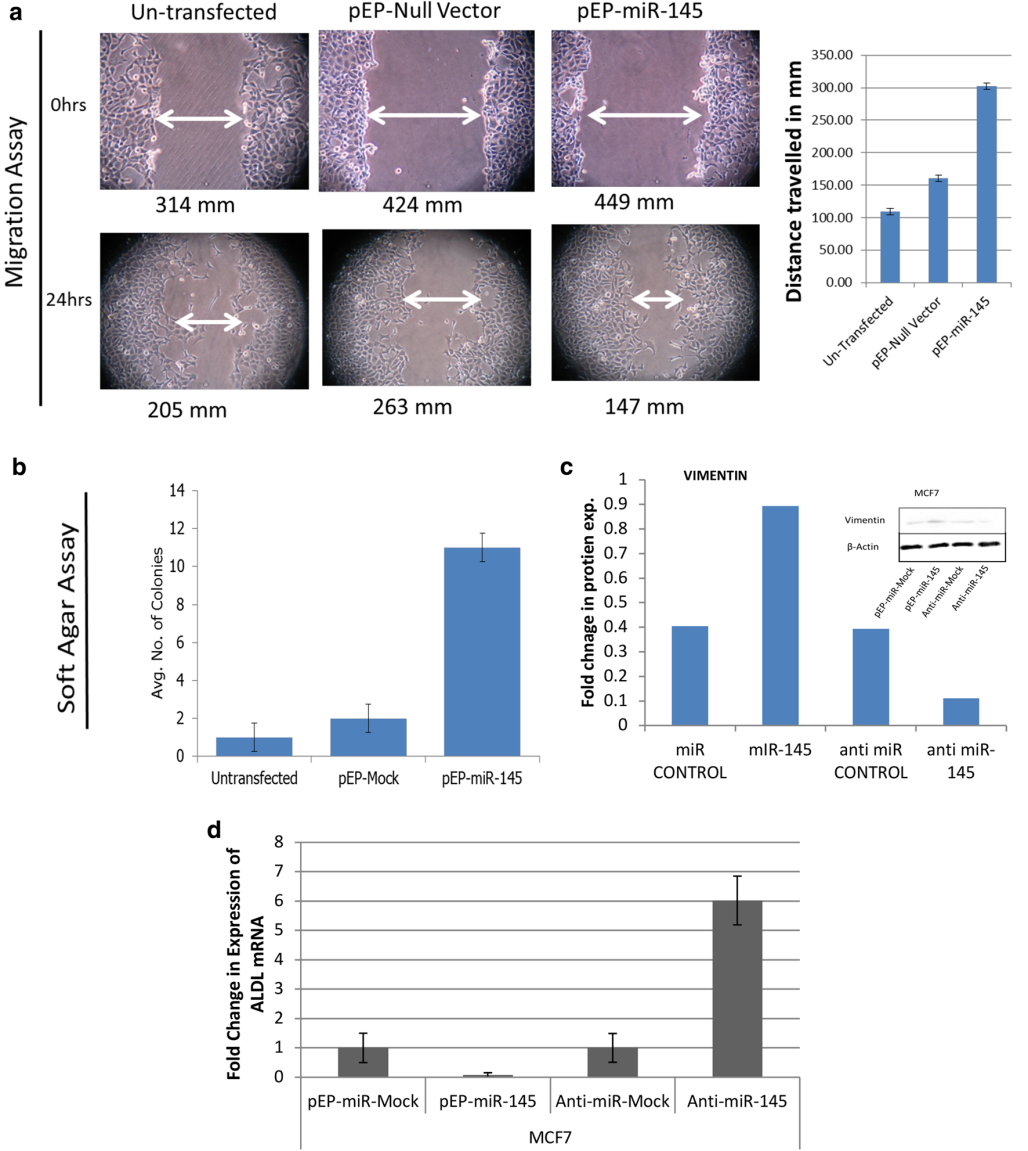
miR-145 over-expression enhanced migration and supported EMT: MCF7 cells (untransfected, pEP-miR-Mock stable and pEP-miR-145 stable) (**a**) were seeded in 6-well plates. 24 h after seeding, a cell free zone was created using sterilized pipette tip and this was defined as 0 h (pre-wounding). Cellular migration in the cell free zone was visualized in pre-wounding wells (0 h) and post-wounding wells (after 24 h). The images were analysed using ImageJ and the distance between two walls of cell free zone (post-wounding–pre-wounding) of miR-145 over-expressing stable cells were found to be significantly decreased as compared to mock transfected stable cells; **b** cells cultured in soft agar and on 14th day fixed and stained using coomassie blue. The miR-145 over-expressing stable cells formed defined colonies suggesting their role in colony formation; **c** Western blots of Vimentin over-expression and down-regulation, and real-time PCR of ALDL mRNA down-regulation and over-expression upon miR-145 over-expression and inhibition, respectively, supporting the positive influence of miR-145 on EMT, in MCF7 cells

The wound healing assay using MCF7 cells showed that the average migration in: (i) untransfected cells—109 ± 10 mm (pre-wounding: 314 ± 10 mm; post-wounding: 205 ± 10 mm); (ii) pEP-miR-Mock cells—161 ± 20 mm (pre-wounding: 424 ± 20 mm; post-wounding: 263 ± 21 mm); and (iii) pEP-miR-145 transfected cells—302 mm ± 15 mm (pre-wounding: 449 ± 17 mm; post-wounding: 147 ± 19 mm) (Fig. 5a), supported cellular migration due to miR-145 over-expression. Further, the number of colonies in soft agar when counted under each condition, showed on an average: only 1 ± 1 colony in un-transfected MCF7 cells, 2 ± 1 colonies in pEP-miR-Mock cells and 11 ± 2 colonies in pEP-miR-145 cells (Fig. 5b). These findings supported the role of miR-145 over-expressing cells in acquiring an apparent anchorage independent growth potential. The miR-145 over-expressing cells, when assessed for the status of EMT markers-Vimentin and *ALDL*, showed a direct correlation. Vimentin expression increased with miR-145 up-regulation, and decreased upon down-regulation of miR-145 (Fig. 5c). Real-time PCR analysis of the expression of *ALDL* showed a decrease (0.2-fold) and increase (sixfold) in presence and absence of miR-145 (Fig. 5d), respectively, as compared to mock (pEP-miR-Mock and anti-miR-Mock considered as onefold, respectively). Such a profile specific to EMT was suggestive of the involvement of miR-145 in epithelial to mesenchymal transition.

## Discussion

The targets of miR-145 identified bio-informatically and validated in cellular assays using both precursor and anti-miR-molecules belonged to different cellular pathways. The commonest feature that emerged in the path-way biology between the three targets, SMAD3, DR5, BRCA2, was of regulation of cellular proliferation despite genomic instability.

It was obvious that down-regulation of the two targets, SMAD3 and DR5, which are known to inactivate TGF-β, inhibiting apoptotic pathway [41] and compromising extrinsic apoptotic induction [44], would result in anti-apoptotic state of the cells, supporting cell survival. Whereas, BRCA2, involved in maintaining genomic stability of the cells [42], when down-regulated with miR-145, was expected to result in accumulation of DNA damage [43]. Anti-apoptosis taken together with DNA damage has been reported to create genomic instability [44] which leads to uncontrolled cellular proliferation like characteristics [45], with a potential role in cancer. This role of miR-145 in facilitating cell proliferation and survival, as observed in wound healing assay and the features associated with epithelial–mesenchymal transition (EMT) (Fig. 5), found support in the epigenetic study of a select set of apoptotic and DDR genes in a representative set of sporadic breast tumours (Figs. 3, 4). Here, in stage 3 and 4 tumours, not only was the expression of miR-145 high (Fig. 2) but it showed similar features of induction of epigenetic imprint in the studied genes, as was observed in miR-145 over-expressing cells in vitro (Figs. 3, 4). These findings along with the bio-informatics pathway analysis, identifying cross talk with molecules involved in growth and EMT (Additional file 2: Figure S1), supported that regulation of SMAD3, DR5 and BRCA2 by miR-145 plays a significant role in uncontrolled cellular proliferation with relevance in cancer.

Earlier studies have reported that metastasis/cellular aggressiveness are accompanied with epithelial to mesenchymal transitions [46]; and the EMT was found to be induced by damaged DNA [47]. Keeping these reports and our observations in view, the epigenetic status of genes involved in survival (anti-apoptotic) and death (apoptotic) pathway was analysed in tumour samples studied earlier [40] and under in vitro miR-145 regulation in the present study. No significant change in expression as well as methylation pattern of CpG positions of death inducing (70 positions) and survival genes (59 positions) was observed in tumour samples (Data not shown) as well as miR-145 expressing (pEP-miR-145) stable cells as compared to mock (pEP-miR-Mock) controls. Similarly, no difference in cellular viability, measured using MTT assay was observed in these cells as well (Additional file 2: Figure S2). However out of 139 CpG positions studied, the average percentage methylation (Additional file 1: Table S1) of only CpG positions of *BCL2*, *TP53*, *RNF8*, *TIP60*, *CHK2*, *DCR2* (Figs. 3, 4) showed association with expression status of miR-145. We were able to observe 6 CpG positions where methylation increased (Fig. 3) and 4 CpG positions where methylation decreased (Fig. 4), under the influence of increased expression of miR-145, both in vivo (tumours) and in vitro conditions. Such hyper (*DR5*, *BCL2*, *TP53*, *RNF8*) and hypo (*CHK2*, *DCR2*) methylation supported the observation of miR-145 being pro-proliferative in nature. These positions were subjected to in silico transcription factor analysis (Using Alibaba 2.2). It was observed that methylation in DR5 was able to create RAP1 binding site, where an elevated RAP1 is known to regulate the activity of E-Cadherin [48], a hallmark for EMT [49]. An overall methylation in TP53 resulted in the creation of a new binding site for OCT-1. Accumulation in OCT-1 has also been associated with unregulated EMT [50–52]. These observations have added strength to our findings of association of miR-145 with EMT pathway. To sum up, we identified SMAD3, DR5 and BRCA2 as novel targets for miR-145. Apart from DR5–3′UTR being the target of miR, we also identified the increase in methylation of *DR5*, *BCL2*, *TP53*, *RNF8* and concomitant hypo methylation in *DCR2*, and *CHK2* promoter regions in presence of miR-145.

## Conclusion

In conclusion, our study identified SMAD3, BRCA2 and DR5 as novel targets of miR-145 and observed the epigenetic association between the increased expression of microRNA and the differential epigenetic regulation of apoptotic and DDR gene related cellular machinery. Keeping in view the observations made, both in vivo and in vitro, we conclude that miR-145 has a pro-proliferative role in late stage node positive tumours and in regulating EMT transition. The association between miR-145 over-expression and differential methylation patterns in in vitro studies and stage 3 + 4 node positive tumours in the representative target genes (*DR5*, *BCL2*, *TP53*, *RNF8*, *TIP60*, *CHK2*, and *DCR2*) supports these inferences.

## Materials and methods

### Bio-informatics predictions

The targets for miR-45 were predicted using three target prediction program (miRanda 3.3a [53], TargetScan 6.2 [54] and RNAhybrid 2.1 [55]). Fasta sequence of hsa-miR-145 was retrieved from miRBase. The binding site in the predicted targets for miR-145 was scanned using default parameters in all three software’s. 3′UTR sequences of SMAD3, BRCA2 and DR5 genes were retrieved from UCSC genome table browser.

### Cloning

miR-145 expressing vectors were generated using pre-miR amplicon of miR-145 from genomic DNA and pEP-miR vector (Cell Biolabs) as backbone. The pre-miR sequence was obtained using primer sets (Additional file 3: Table S2) and amplified from human genomic DNA by simple PCR reaction. The primers (IDT) were designed using online (Primer 3) and offline (Oligo) tools such that the 5′ ends of each forward and reverse primer contained *Nhe*-I and *Bam*H1 restriction sites, respectively. These sites were also identified in pEP-miR vector for cloning purposes. After amplification the product was digested and cloned into pEP-miR vector, generating pEP-miR-145 recombinant clone. For anti-miR conditions; commercially available controls and inhibitor molecules were purchased (Applied Biosystems) and used.

### Luciferase assay

The 3′UTR of novel targets, established bio-informatically, were cloned into the 3′UTR of pGL3-Control vector (Promega) at *Xba*-I site. The 3′UTR was amplified from human genomic DNA, using primer (IDT) sets (Additional file 3: Table S2). The amplicons were then cloned, generating pGL3-SMAD3, pGL3-BRCA2 and pGL3-DR5 vectors. All the clones were verified for containing the desired insert, by colony PCR, restriction digestion and DNA sequencing. Both pGL3 control and pEP-miR-145 (The miR-145 expressing vector) constructs were co-transfected in two different cell lines, MCF7 and HepG2. The transfection was performed using ESCORTS (Sigma-Aldrich) reagent and according to manufacturer’s protocol. The assay was performed after seeding in MCF7 and HepG2 cells, in a 24-well plate, using Dual-Luciferase Assay Kit (Promega). After 48 h of transfection the cells were measured for Firefly and Renila luminescence, using luminometer. The ratio of Firefly and Renila reporter in presence of pEP-miR-145 was calculated and used in defining the change in expression of Firefly reporter in co-presence of predicted binding sites of specific genes.

### Wound-healing assay

To begin with the untransfected, pEP-miR-Mock and pEP-miR-145 transfected cells were seeded in 6-well plates. A cell free zone was created, 24 h after seeding, using a pipette tip. This was termed as pre-wounding well (0 h). These cells were allowed to grow for 24 h (post-wounding). These cells were visualized and images were captured. The images were analysed using ImageJ software and the distance between the cellular walls of pre-migration and post-migration were measured.

### Generation of stable cells

pEP-miR-145 and pEP-Mock (Vector containing Anti-puromycin gene) were transfected independently in MCF7 cells using ESCORT (Sigma-Aldrich) reagent. The transfected cells were grown in media containing puromycin as selection marker. After seven passages the cells were verified for transient expression of miR-145 before further experimentation was performed. Thereafter, these sets of miR-145 expressing and pEP-mock transfected cells were cultured in puromycin containing media for all experimentation.

### Soft-Agar assay

miR-145 expressing and pEP-Mock stable cells were re-suspended in 2-ml 0.3% agar medium. Each embedded cell mixture was overlaid on 1.5 ml, 0.6% agar in 6-well plates, and a 1.5-ml top layer of 0.6% agar added to each well to prevent evaporation. The cultures were incubated in a humidified incubator at 37 °C and 5% CO_2_ for about 2 weeks. Cell colonies were stained with giemsa and counted using a conventional microscope [56].

### Western blotting

Cell lysate were prepared by incubating cells in buffer containing 0.5% sodium deoxycholate, 1% Triton X-100, 50 mM Tris pH 7.2, 150 mM NaCl, 10% glycerol, 0.1% SDS, 1 mM dithiothreitol (DTT), 1 mM sodium vanadate (NaV), 1 mM phenylmethylsulfonyl fluoride (PMSF), 5 mM sodium fluoride (NaF), phosphatase inhibitor cocktail (Sigma), 4 µg/ml each of pepstatin, aprotinin and leupeptin (Sigma), on ice for 30 min. Concentration of isolated protein was estimated using Pierce BCA (bicinchoninic acid) protein assays as per the manufacturer protocol. Separation of proteins was done on 10% SDS-PAGE, later transferred to nitrocellulose membrane (mdi) overnight at 4 °C (wet transfer), and probed with primary antibodies. Membrane was incubated with appropriate secondary antibody for 1 h at room temperature. Proteins were detected using enhanced chemiluminescence kit from Thermo Scientific, USA. Primary antibodies used were: anti-vimentin and anti-β-actin (Cell signaling Technology).

### Real-time PCR

Total RNA was extracted using traditional TRIzol (Sigma) method. RNA quality was analyzed by electrophoresis on 1.2% agarose formaldehyde gel and quantified by A260/A280 absorbance equilibrium. 1–2 µg of total RNA was reverse transcribed into single stranded DNA using High-Throughput cDNA preparation kit (Applied Biosystems). Sybr-Green assay (Applied Biosystems) was used for quantitating mRNA levels of ALDL. GAPDH was used as endogenous control. Real time PCR was carried out on ABI Prism 7000 Sequence Detection System (Applied Biosystem). ΔΔCt (Cycle threshold) method of relative quantification was used to calculate fold change in gene expression by SDS 1.1 RQ software (Applied Biosystems).

### Methylation analysis

CpG positions (nomenclature as per transcription start site (TSS)) of apoptosis and DDR pathway genes were selected and identified as per earlier study [40]. The EZ-96 DNA Methylation Kit (Zymo Research) was used for bisulfite conversion of the target sequences. PCR products were analyzed further in Sequenom MassARRAY.

### Statistical analysis

Spearman’s correlation coefficient and Fisher’s exact test was calculated using the SPSS version 13. p value at and below ≤ 0.05 was considered significant.

## Supplementary information

**Additional file 1: Table S1.** Percentage methylation values of 10 CpG positions: identified to be up regulated or down regulated in association with miR-145 expression under both in-vitro (Mock, miR-145) and in*-*vivo (Stage 1 + 2, Stage 3 + 4, Node 0, Node 1, Node 2, Node 3) and conditions.

**Additional file 2: Figure S1.** Bio-informatics prediction of cross-talk between miR-145, SMAD3, BRCA2 and DR5. **Figure S2.** miR-145 constrains mRNA expression of death inducing genes: Comparison of (a) the average pooled mRNA expression, by Real-Time PCR, of death inducing (BRCA2, CYC, DR5, MDM2, ALDL, SMAD3, TGFB) Vs survival inducing (FLIPL, BCL2, CASP8L, TP53, PKM2) genes suggests constrained expression of death inducing genes under miR-145 up-regulation as compared to miR-145 inhibition. (b) the average percentage methylation of CpG positions of death inducing genes (70 positions) and survival inducing genes (59 positions), and (c) the cellular viability, under pEP-miR-Mock and exogenous pEP-miR-145 overexpression, suggested no significant change.

**Additional file 3: Table S2.** List of primers used for cloning.

## Data Availability

The material and data defined in the manuscript is available on demand by contacting the corresponding author at pawandhar@mail.jnu.ac.in.

## Abbreviations

EMT: epithelial to mesenchymal transition
MiR: microRNA
DDR: DNA damage response
3′ UTR: 3′ untranslated region
PMSF: phenylmethylsulfonyl fluoride
