# Supplementary material for: miR-145 supports cancer cell survival and shows association with DDR genes, methylation pattern, and epithelial to mesenchymal transition
Source: Cancer Cell Int. 2019 Sep 6;19:230. doi: 10.1186/s12935-019-0933-8 (PMC6731614; doi:10.1186/s12935-019-0933-8)
Supplement: Supplementary file 1 — Additional file 1: Table S1. Percentage methylation values of 10 CpG positions: identified to be up regulated or down regulated in association with miR-145 expression under both in-vitro (Mock, miR-145) and in-vivo (Stage 1 + 2, Stage 3 + 4, Node 0, Node 1, Node 2, Node 3) and conditions. [file 12935_2019_933_MOESM1_ESM.docx]

| Gene Name | CpG Position (Transcription Start Site) | Methylation Status (%) | | | | | | | |
| --- | --- | --- | --- | --- | --- | --- | --- | --- | --- |
|  |  | ***In-vitro*** | | ***In-vivo*** | | | | | |
|  |  | **Mock (pEP-Null)** | **miR-145 (pEP-miR-145)** | **Stage 1 + 2** | **Stage 3 + 4** | **N0** | **N1** | **N2** | **N3** |
| DR5 | (-93,-91) | 12.00 | 13.00 | 2.00 | 2.88 | 1.00 | 1.50 | 2.00 | 5.00 |
| BCL2 | (-598,-591,-589,-586) | 3.00 | 5.00 | -0.83 | 0.41 | -0.66 | 1.25 | 3.50 | 4.00 |
| TP53 | (-78, -75, -22, -15, +91, +158, +171, +175) | 28.00 | 33.00 | 0.05 | 1.43 | 4.50 | -0.67 | -0.04 | 1.17 |
| RNF8 | (+11) | 4.00 | 16.00 | 3.17 | 3.91 | 0.16 | -0.01 | 0.00 | 0.14 |
| RNF8 | (+75, +82) | 2.00 | 7.00 | 1.00 | 3.50 | 0.12 | -0.02 | 0.01 | 0.07 |
| TIP60 | (-10, -3) | 3.00 | 4.00 | -0.17 | -0.08 | -0.25 | -0.50 | -0.20 | 1.00 |
| TIP60 | (-74) | 5.00 | 3.00 | 1.17 | -0.67 | 2.00 | 1.00 | -1.75 | -4.00 |
| CHK2 | (-279, -268) | 6.00 | 2.00 | 0.50 | -1.33 | -0.02 | -0.01 | -0.01 | -0.05 |
| DCR2 | (-263) | 7.00 | 2.00 | 11.17 | 4.08 | 0.13 | 0.04 | 0.03 | 0.00 |
| DCR2 | (-23, -13) | 3.00 | 1.00 | 14.17 | 5.41 | 15.25 | 8.50 | 4.00 | 4.00 |

**Additional Table S1**
