# Supplementary figures and images for: miR-145 supports cancer cell survival and shows association with DDR genes, methylation pattern, and epithelial to mesenchymal transition
Source: Cancer Cell Int. 2019 Sep 6;19:230. doi: 10.1186/s12935-019-0933-8 (PMC6731614; doi:10.1186/s12935-019-0933-8)

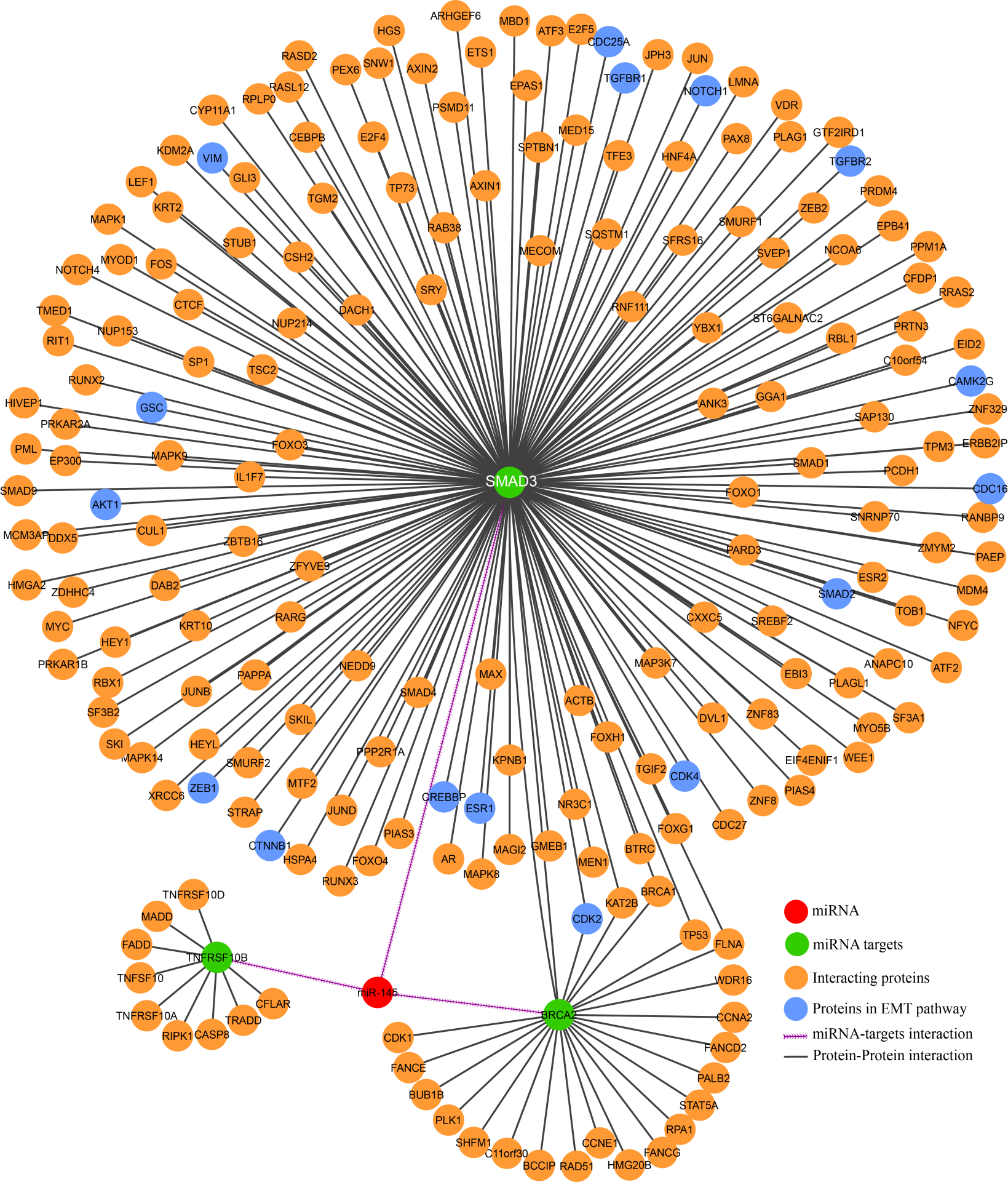


**Additional Figure S1**

(b)

(c)

**Additional Figure S2**

Supplement: Supplementary file 2 — Additional file 2: Figure S1. Bio-informatics prediction of cross-talk between miR-145, SMAD3, BRCA2 and DR5. Figure S2. miR-145 constrains mRNA expression of death inducing genes: Comparison of (a) the average pooled mRNA expression, by Real-Time PCR, of death inducing (BRCA2, CYC, DR5, MDM2, ALDL, SMAD3, TGFB) Vs survival inducing (FLIPL, BCL2, CASP8L, TP53, PKM2) genes suggests constrained expression of death inducing genes under miR-145 up-regulation as compared to miR-145 inhibition. (b) the average percentage methylation of CpG positions of death inducing genes (70 positions) and survival inducing genes (59 positions), and (c) the cellular viability, under pEP-miR-Mock and exogenous pEP-miR-145 overexpression, suggested no significant change. [file 12935_2019_933_MOESM2_ESM.docx]
