# Supplementary material for: miR-145 supports cancer cell survival and shows association with DDR genes, methylation pattern, and epithelial to mesenchymal transition
Source: Cancer Cell Int. 2019 Sep 6;19:230. doi: 10.1186/s12935-019-0933-8 (PMC6731614; doi:10.1186/s12935-019-0933-8)
Supplement: Supplementary file 3 — Additional file 3: Table S2. List of primers used for cloning. [file 12935_2019_933_MOESM3_ESM.docx]

**Table S2: List of primers used for cloning**

| **Gene Name** | **Primer Pairs** | **Tm** |
| --- | --- | --- |
| hsa-miR-145 | Forward:  5’-TCGAGGATCCGGCTGGATGCAGAAGAGAAC -3’  Reverse:  5’-TCGAGCTAGCCAGGGACAGCCTTCTTCTTG -3’ | 60ºC |
| SMAD3 | Forward:  5’-AATCTAGACAGCAGAGAAGCTGTAGGACTG-3’  Reverse:  5’-AATCTAGAGACAGGGACCTGGACATGAG-3’ | 62ºC |
| BRCA2 | Forward:  5’-AATCTAGAAACAAACATCACAGCCCTCAC–3’  Reverse:  5’-AATCTAGACTCACAAATGCCTCAGCAAA–3’ | 62ºC |
| DR5 | Forward:  5’-TCGAGGATCCGCTGGGCTCTGATCCTTCTT -3’  Reverse:  5’-TCGAGCTAGCCACCAACAACTACCCCATGTT -3’ | 62ºC |
